# HC-HA/PTX3 from amniotic membrane reprograms human corneal fibroblasts to neural crest progenitors by switching from canonical to noncanonical TGFβ signaling

**DOI:** 10.1186/s13287-026-04983-w

**Published:** 2026-03-21

**Authors:** Ying-Ting Zhu, Sean Tighe, Yuan Zhang, Allison Helman, Scheffer C. G. Tseng

**Affiliations:** R&D Department, BioTissue Holdings Inc., 7300 Corporate Center Drive, Miami, FL 33126 USA

**Keywords:** Amniotic membrane, Cyclin D1, Fibroblast, HC-HA/PTX3, Myofibroblast, Neural crest, TGFβ1, TAK1, Reprogramming

## Abstract

**Background:**

HC-HA/PTX3 (a complex formed by high molecular weight hyaluronan covalently linked to heavy chain 1 of inter-α-trypsin inhibitor and tightly bound to pentraxin 3) is a unique extracellular matrix from human amniotic membrane that exerts an anti-scarring action and reprograms human corneal fibroblasts (HCF) and myofibroblasts to corneal stromal keratocytes in the absence of transforming growth factor β1 (TGFβ1) by downregulating canonical Smad-mediated signaling and upregulating bone morphogenetic protein (BMP) signaling. It remains unclear whether HC-HA/PTX3 can further reprogram HCF into neural crest (NC) progenitors in the presence of TGFβ1.

**Methods:**

Human corneal fibroblasts were seeded on plastic, immobilized hyaluronic acid (HA) or HC-HA/PTX3 or on plastic with or without soluble HA and HC-HA/PTX3 in DMEM + 10% fetal bovine serum (FBS), with or without various inhibitors with or without TGFβ1. Transcript expression of NC and signaling markers was determined by RT-qPCR. Immunostaining was performed to monitor cytolocalization of signaling markers and α-smooth muscle actin (α-SMA). Raft separation before Western blot was used to study protein distributions in both rafts. Western blot and ELISA were used to measure relative protein level.

**Results:**

Herein, we show for the first time that in the presence of exogenous TGFβ1, HC-HA/PTX3 continues to reprogram HCF into NC progenitors by upregulating mRNA expression of NC markers, confirmed by successful induction into human corneal endothelial cells, highlighted by hexagonal shape, mRNA expression of corneal endothelial markers and junctional staining of corneal endothelial markers such as Na-K-ATPase, α-catenin, β-catenin, F-actin, N-cadherin, p120 and ZO-1 but the lack of fibrogenic marker S100A4. Such reprogramming requires suppression of TGFβ1 SMAD-mediated canonical signaling that starts from HC-HA/PTX3 binding with CD44 to sequester type II TGFβ receptor (TβRII) in lipid raft and ends with downregulation of TβRII by nuclear translocation of cyclin D1. Mechanistically, nuclear translocation of cyclin D1 is mediated by activation of transforming growth factor-beta-activated kinase 1-transcription factor Jun (TAK1-cJUN) noncanonical signaling because of type I TGFβ receptor (TβRI) and type III TGFβ receptor (TβRIII) (without TβRII) in non-lipid raft as well as by nuclear translocation of CD44 intracellular domain (CD44ICD) formed by Membrane Type 1 Matrix Metalloproteinase (MT1MMP)/γ-secretase cleavage to facilitate such reprogramming.

**Conclusions:**

Thus, HC-HA/PTX3 from amniotic membrane can be deployed as a new strategy to reverse scar toward regeneration.

**Supplementary Information:**

The online version contains supplementary material available at10.1186/s13287-026-04983-w.

## Background

In the cornea, both stromal keratocytes and endothelial cells are derived from cranial neural crest (NC) progenitors [1, 2] and help maintain corneal transparency by providing an organized lamellar extracellular matrix and exerting pump and barrier functions, respectively (reviewed in [3, 4]). Under pathological conditions, both keratocytes and endothelial cells may differentiate into fibroblasts and α-smooth muscle actin (α-SMA)-expressing myofibroblasts, leading to scar formation and corneal blindness [5].

Transforming growth factor β1 (TGFβ1) is a major growth factor promoting the aforementioned pathogenic differentiation of keratocytes and corneal endothelial cells into fibroblasts and myofibroblasts [6–9]. TGFβ1 together with TGFβ2 and TGFβ3 (an anti-scarring isoform [10]) are ligands in the TGFβ family [11] that interact with three receptors, i.e., type I TGFβ receptor (TβRI), type II TGFβ receptor (TβRII) and type III TGFβ receptor (TβRIII) [12]. In canonical signaling pathway, TGFβ1 binds to TβRII, which recruits TβRI that then phosphorylates SMAD2/3 for nuclear translocation to modulate gene transcription [13, 14]. TGFβ1-mediated canonical signaling is involved in scar formation characterized by upregulation of collagens and proteoglycans, downregulation of proteinase and matrix metalloproteinases (MMP), and upregulation of their inhibitors [15, 16] and α-SMA expression [17–20]. TGFβ1 may also activate noncanonical (SMAD-independent) signaling through mitogen-activated protein kinase (MAPK) pathways including extracellular signal-regulated kinase (ERK), transforming growth factor-beta-activated kinase 1 (TAK1), p38 MAPK, and Jun N-terminal kinase (JNK), phosphoinositide 3-kinase-Akt-mTOR pathway, and GTPases Rho, Rac, and Cdc42 pathways [21–23]. It remains puzzling how TGFβ noncanonical signaling seemingly participates in conflicting outcomes, i.e., augmentation of scar formation [24, 25] versus tissue regeneration [26–29].

Clinical transplantation of human amniotic membrane (AM) prevents scar formation in a number of diseases [30–32]. Mechanistically, AM’s anti-scarring action involves downregulation of TGFβ1-mediated SMAD signaling [33–36]. We have demonstrated that such anti-scarring action is retained in AM soluble extract [35] from which HC-HA/PTX3 has been purified and characterized as a complex formed by high molecular weight hyaluronan covalently linked to heavy chain 1 of inter-α-trypsin inhibitor and tightly bound to pentraxin 3 (PTX3) [37, 38]. HC-HA/PTX3 is the prime candidate responsible for AM’s anti-scarring action because it does not only suppress the TGFβ1 promoter activity in human corneal fibroblasts (HCF) cultured on plastic [37] but also reprograms HCF and myofibroblasts into keratocytes in the absence of TGFβ1 [35]. Such reprogramming was initiated by cell aggregation mediated by C-X-C motif chemokine receptor 4 (CXCR4) signaling able to be blocked by AMD3100 followed by activation of bone morphogenetic protein (BMP) canonical signaling [35]. Herein, we extend our study to show that HC-HA/PTX3 continues to reprogram HCF to NC progenitors in the presence of TGFβ1 by switching to noncanonical TAK1- transcription factor Jun (cJUN) signaling and by activating nuclear translocation of CD44 intracellular domain (CD44ICD). Therefore, HC-HA/PTX3 from AM may not only prevent scar formation but also reverse scar toward regeneration by reprogramming to progenitors even in the presence of pro-fibrotic TGFβ1.

## Methods

### Materials

All materials used for assays, cell culture, and manipulation are listed in Table S1. All primers used for quantitative real time polymerase chain reaction (qRT-PCR) are listed in Table S2. All antibodies used for immunofluorescence staining and Western blot are listed in Table S3.

### Purification and immobilization of HC-HA/PTX3

HC-HA/PTX3 was purified from cryopreserved AM procured by BioTissue Holdings, Inc. (Miami, FL) using donated human placenta after donor eligibility was determined according to Good Tissue Practice (GTP) set forth by the FDA and following our published method with modifications [37, 39]. In brief, AM was cryopulverized under liquid nitrogen by FreezeMill (FreezerMill 6870, SPEX^®^ SamplePrep, Metuchen, NJ), extracted by PBS (pH 7.4) at 4 °C for 1 hour (h) and then centrifuged at 48,000×*g* at 4 °C for 30 minutes (min) to generate water-soluble AM extract. Solid cesium chloride (CsCl) and 8 M Guanidine-HCl/PBS (GnHCl) with protease inhibitors were added and mixed at room temperature to achieve 4 M GnHCl, a density of 1.35 g/ml of CsCl, and a final concentration of 10 mM aminocaproic acid, 10 mM EDTA, 10 mM N-ethylmaleimide, and 1 mM phenylmethylsulfonyl fluoride before centrifugation at 125,000×*g* at 15 °C for 66 h (SW 32 Ti Swinging-Bucket Rotor, Beckman Coulter, Inc., Brea, CA). Each of the 12 fractions (3 ml/fraction) from top to bottom per tube was measured for HA and protein contents by the enzyme-linked immunosorbent HA Quantitative Test Kit and the BCA Protein Assay Kit, respectively. After excluding fractions 1 and 2, which contained proteins without HA, the remaining fractions were pooled and further subjected to three consecutive runs of ultracentrifugation at 125,000*g* in CsCl/4 M guanidine HCl at a density of 1.40 g/ml for the 2nd and 1.42 g/ml for the 3rd and 4th run, each run at 15 °C for 48 h. After the fourth ultracentrifugation, fractions 3 to 9 were pooled, dialyzed (dialysis tube, 3 kD MWCO) extensively against filtered water, lyophilized (SP Scientific AdVantage Pro XL Freeze Dryer/Lyophilizer, SP, Warminster, PA), and stored at -80 °C before use with a validated shelf life of 4 years.

Before release for use, HC-HA/PTX3 purity was verified by the lack of detectable proteins per the BCA assay with a detectable level of 11.7 ± 3.2 µg/ml. Due to the lack of detectable proteins, HC-HA/PTX3 was quantified per HA amount. Each batch of HC-HA/PTX3 was released for use after meeting the acceptance criteria of HA concentration no more than (NMT) 50.12 µg/ml (upper control limit) and HA in HC-HA/PTX3 was of high molecular weight (HMW) (≥ 500 kDa) using agarose gel electrophoresis [40]. The identity of HC-HA/PTX3 was verified by Western blot analysis using respective antibodies specific to HC1 and PTX3 with or without hyaluronidase (HAase) digestion to release HC1 and HMW PTX3 from HC-HA/PTX3 in the loading well and with or without reduction by DTT, which further rendered PTX3 from HMW including octamer to dimer and monomer [40]. Finally, each batch of HC-HA/PTX3 was released after passing a potency assay by exhibiting no less than (NLT) 90% of inhibiting the tartrate-resistant acid phosphatase (TRAP) activity of osteoclast differentiation in cloned monocytes of murine RAW264.7 cell line (ATCC, Manassas, VA) promoted by receptor activator of nuclear factor kappa-Β ligand (RANKL) [40].

As reported [41], HC-HA/PTX3 or high molecular weight HA (each at 2 µg/well) was immobilized by mixing it with Sulfo-NHS at 9.2 mg/ml and 1-ethyl-3-(3-dimethylaminopropyl) carbodiimide at 6.15 mg/ml in each well (100 µl) followed by incubation at 4 °C overnight. Un-crosslinked HC-HA/PTX3 and crosslinking reagents were removed by washing twice with 2 M NaCl/50 mM MgSO_4_/PBS, followed by two washes of PBS. After confirming that mRNA expression of NC markers and cytoplasmic location of p75 neurotrophin receptor (p75NTR) and cyclin D1 noted in HCF cultures on immobilized HC-HA/PTX3 without TGFβ1 was replicated by use of soluble HC-HA/PTX3 (not shown), lyophilized HC-HA/PTX3 was reconstituted in culture media and added directly to HCF cultures as soluble HC-HA/PTX3 in experiments to further decipher signaling mediated by HC-HA/PTX3.

### Isolation and culture of HCF

Human corneas from donors aged 18–76 years maintained at 4 °C in Optisol (Chiron Vision, Irvine, CA) for less than 7 days after death were obtained from the Florida Lions Eye Bank (Miami, FL) and handled according to the declaration of Helsinki. HCF were isolated from donor corneas and cultured as reported [42]. After peeling off the endothelium by forceps and removing the epithelium by digestion with 10 mg/ml dispase at 4 °C overnight, the remaining corneal stroma was cut into cubes of approximately 1 mm^3^, incubated in 2 mg/ml collagenase for 16 h at 37° C, and then placed on plastic of a 6-well dish in DMEM supplemented with 10% fetal bovine serum containing 50 mg/ml gentamicin and 1.25 mg/ml amphotericin B (DMEM + 10%FBS). The culture medium was replenished twice a week. HCF at 70% confluence were passaged at 1 to 3 ratio using 0.25% trypsin and expanded in DMEM + 10%FBS until passage 3 (P3) used for experiments.

### Treatment of HCF culture

To test cellular responses to TGFβ1 on immobilized HC-HA/PTX3 or HA, P3 HCF (15,000/cm^2^) were seeded in DMEM + 10%FBS for 72 h and then switched to DMEM + ITS for 24 h before being treated with or without 10 ng/ml of TGFβ1. Cell morphology was documented by phase-contrast microscopy at day 1, 3, 5 and 7. Cells were harvested at 24 h for mRNA expression levels of TGFβs, TβRs, keratocan, a marker for keratocytes [35] and embryonic stem cell (ESC) and NC markers including p75NTR, and immunostaining of pSMAD2/3, p75NTR and cyclin D1, at 48 h for ELISA measurement of TGFβ proteins and Western blot for keratocan, p75NTR and cyclin D1 proteins using β-actin as the control, and at 72 h for immunostaining of α-SMA, a marker of myofibroblasts [43].

To confirm NC progenitors reprogrammed by HC-HA/PTX3, P3 HCF cultured on plastic at a density of 20,000 cells/24-well with or without immobilized HC-HA/PTX3+TGFβ1 (10 ng/ml) in DMEM + ITS for 24 h were switched to low-glucose, low-calcium DMEM + 10% FBS for 3 weeks, a method used to induce human NC progenitors into human corneal endothelial cells (HCEC) [44]. The HCEC control was isolated from donor corneas obtained from the Florida Lions Eye Bank (Miami, FL) as described previously [45–47]. In short, the corneoscleral rims were rinsed three times with DMEM containing 50 µg/ml gentamicin and 1.25 µg/ml amphotericin B. Under a dissecting microscope, the trabecular meshwork was cleaned up to the Schwalbe’s line. The remaining corneal rim from which the Descemet’s membranes was stripped was digested at 37 °C for 16 h with 1 mg/ml collagenase A in MESCM, which was made of DMEM/F-12 (1:1) supplemented with 10% knockout serum, 5 µg/ml insulin, 5 µg/ml transferrin, 5 ng/ml sodium selenite, 4 ng/ml basic fibroblast growth factor (bFGF), 10 ng/ml human leukemia inhibitory factor (hLIF), 50 µg/ml gentamicin, and 1.25 µg/ml amphotericin B plus 5% FBS. The resultant aggregates of HCEC with undigested basement membrane matrix were collected by centrifugation at 1000 rpm for 3 min to remove the digestion solution and cultured in 24-well dishes coated with collagen IV in MESCM plus 5% FBS. mRNA expression and cyto-location of HCEC markers were determined by RT-qPCR and immunostaining, respectively.

Because the TβRII promoter contains an inverted CCAAT box [48] and cyclin D1 has been shown to inhibit CCAAT box-containing enhancer binding proteins [49, 50], the role of cyclin D1 was elucidated by downregulating cyclin D1 by 100 nM cyclin D1 siRNA in P3 HCF (15,000/cm^2^) cultured on immobilized HC-HA/PTX3 (20 µg/ml) in DMEM + ITS+TGFβ1 (10 ng/ml) for 24 h to investigate transcript expression of TβRs, cyclin D1 and NC markers and immunostaining of pSMAD2/3, cyclin D1 and p75NTR, Western blot analysis of TβRs, cyclin D1, p75NTR with or without cyclin D1 siRNA and β-actin as the loading control at 48 h, and immunostaining of α-SMA with or without cyclin D1 siRNA at 72 h.

The involvement of CD44 receptor for binding HC-HA/PTX3 was probed by pretreatment with α-CD44 blocking antibody (Table S3) or isoantibody (10 µg/ml each) for 30 min in P3 HCF cultured on plastic in DMEM + ITS before being treated with or without soluble HC-HA/PTX3 or HA (20 µg/ml each)+TGFβ1 (10 ng/ml) with or without α-CD44 antibody using isoantibody as the control for 0 to 5 min before membrane and cytoplasmic preparation was precipitated with CD44 antibody (Ab254530) and Western blot to study interaction between CD44 and TβRs. The above cultures were also harvested for lipid raft and non-lipid raft separation before Western blot using antibodies against CD44 and TβRs to study their distributions in both rafts.

To screen various kinases possibly involved in TGFβ noncanonical signaling, P3 HCF seeded on plastic in DMEM + 10% FBS for 72 h were switched to DMEM + ITS for 24 h, then treated with or without soluble HA (20 µg/ml) or HC-HA/PTX3 (20 µg/ml) ± TGFβ1 (10 ng/ml) with or without α-CD44 antibody using isoantibody as the control for 0 and 5 min before being harvested for immunostaining and Western blot for various phospho-kinases using β-actin as the loading control.

Because unique downregulation of TβRII and suppression of nuclear translocation of pSMAD2/3, TAK1-cJUN-cyclin D signaling was investigated by addition of TAK1 inhibitor (5*Z*)-7-Oxozeaenol (20 nM) for 0, 1, 3 and 5 min before Western blot of pTAK1 and pcJUN, for 0, 5, 30 and 45 min before immunostaining of pcJUN, cyclin D1 and p75NTR, for 0 and 24 h before qRT-PCR of cyclin D1 and NC markers, and for 0 and 48 h before Western blot of cyclin D1 protein.

The role of CD44ICD was assessed in P3 HCF seeded on glass in DMEM + 10% FBS for 24 h and in DMEM + ITS for 24 h before being treated with/without PBS or HA or HC-HA/PTX3 ± TGFβ1 (10 ng/ml) for 0 and 5 min before lipid raft separation and Western blot using antibodies against Membrane Type 1 Matrix Metalloproteinase (MT1MMP) and catalytic domain of MT1MMP (active MT1MMP) using caveolin-1 and transferrin as lipid and non-lipid raft controls, respectively. After confirming the involvement of MT1MMP, the above cultures were also treated with or without Marimastat (10 µM) (MMP inhibitor) or DAPT (10 µM) (γ-secretase inhibitor) or both for 0 and 5 min for membranous, cytoplasmic and nuclear preparation using NE-PER Nuclear and Cytoplasmic Extraction Reagents (ThermoFisher Scientific, Waltham, MA) before Western blot of nuclear pSMAD2/3, CD44ICD, active MT1MMP and active γ-secretase, and for 0, 5, 15, 30 and 45 min for immunostaining of CD44ICD, cyclin D1 and p75NTR.

### RNA extraction, reverse transcription, and qPCR

Total RNAs were extracted using RNeasy Mini Kit and reverse-transcribed using High-Capacity Reverse Transcription Kit. cDNA was amplified by real-time RT-PCR using specific primers and PCR Master Mix in Quant Studio 5 Real-time PCR System (ThermoFisher Scientific, Waltham, MA). Real-time PCR consisted of 10 min of initial activation at 95 °C, followed by 40 cycles of 15 second (s) denaturation at 95 °C, and 1 min annealing and 1 min extension at 60 °C. The genuine identity of each PCR product was confirmed by the size determination using 2% agarose gel followed by ethidium bromide staining together with PCR marker according to EC3 Imaging System (BioImaging System, Upland, CA).

### Immunofluorescence staining

HCF were harvested with 0.25% trypsin and 1mM EDTA at 37 °C for 5 min and prepared for cytospin using Cytofuge (StatSpin Inc., Norwood, MA) at 1000 rpm for 4 min. Cells were fixed with 4% formaldehyde, pH 7.0, for 15 min at room temperature, permeabilized with 0.2% Triton X-100 in PBS for 30 min and blocked with 2% BSA for 1 h before being incubated with primary antibodies for 16 h at 4 °C. After 3 washes with PBS, the second antibodies were incubated for 60 min at room temperature and followed with the corresponding Alex Fluor-conjugated secondary IgG. Corresponding mouse and rabbit sera were used as negative controls for primary monoclonal and polyclonal antibodies, respectively. The nucleus was counterstained with Hoechst 33,342 before being analyzed with Zeiss LSM 700 confocal microscope (Carl Zeiss, Thornwood, NY).

### TGFβ ELISA

HCF cultures treated with or without TGFβ1 (10 ng/ml) for 24 h were switched to a fresh medium without TGFβ1 for 24 h to eliminate exogenous TGFβ1 before the supernatant was collected for TGFβ1 ELISA using Quantikine Human TGFβ1 Kit. The supernatant of HCF cultures treated, with or without TGFβ1 (10 ng/ml) for 48 h was collected for TGFβ2 and TGF-β3 ELISA using Quantikine Human TGFβ2 ELISA Kit and TGFβ3 ELISA Kit, respectively.

### Western blot

Cell lysates and nuclear extracts were prepared in RIPA buffer, the protein concentrations determined by the BCA Protein Assay Kit (Table S1), and 10 µg of protein for each sample resolved on 4–15% (w/v) gradient acrylamide gels under denaturing conditions for Western blot. The protein extracts were transferred to the nitrocellulose membrane, then blocked with 2% BSA in TBST [50 mM Tris-HCl, pH 7.5, 150 mM NaCl, 0.05% Tween-20 (v/v)], followed by sequential incubation with the specific primary antibodies and its respective horseradish peroxidase (HRP)-conjugated secondary antibodies using β-actin (for cell membrane and cytoplastic protein), histone (for nuclear protein), caveolin-1 (for lipid raft protein) and transferrin (for non-lipid raft protein) as the loading controls. Immunoreactive proteins were detected with Western Lighting Chemiluminescence.

### Statistical analysis

All summary data were reported as means ± S.D. calculated for each group and compared using ANOVA and the Student’s unpaired t-test by Microsoft Excel (Microsoft, Redmont, WA). Test results were reported as two-tailed p values, where *p* < 0.05 was considered statistically significant.

## Results

### HC-HA/PTX3 prevents myofibroblast differentiation under TGFβ1 stimulation and reprograms HCF to NC progenitors

**Fig. 1:**
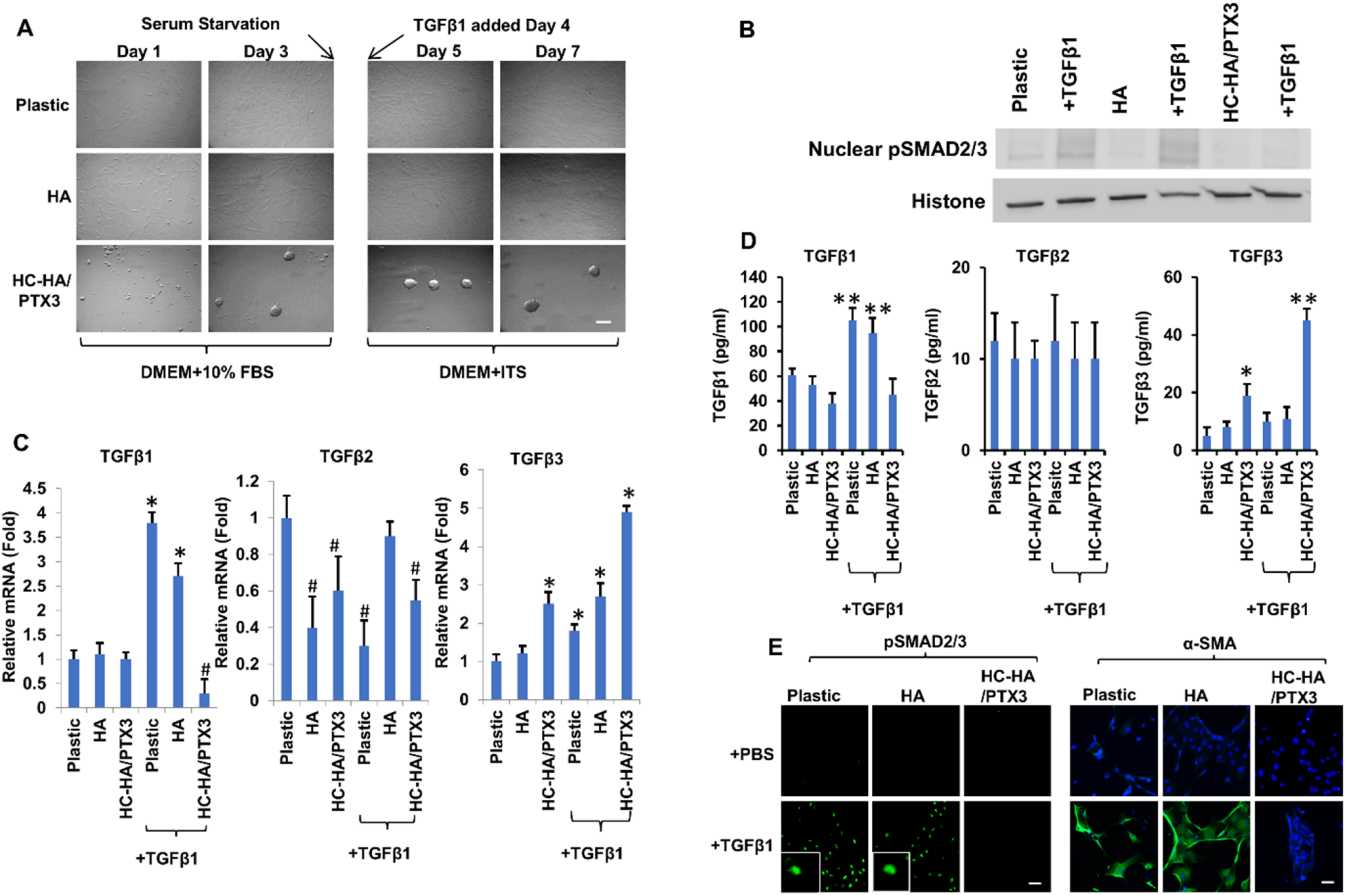
HC-HA/PTX3 suppresses myofibroblast differentiation through inhibition of TGFβ canonical signaling and TGFβ1 autoinduction and upregulation of TGFβ3. P3 HCF seeded on plastic with or without immobilized HA or HC-HA/PTX3 in DMEM + 10% FBS for 72 h were switched to DMEM + insulin-transferrin-selenium (ITS) for 24 h before being added with or without TGFβ1 for up to 72 h (A, Bar = 100 μm). Nuclear fraction was collected 24 h before Western blot of pSMAD2/3 (B, histone as the loading control) and for qRT-PCR of TGFβs transcripts using the expression level on plastic set as 1 (C, *p < 0.05, **p < 0.01, ^#^*p* < 0.05, downregulated, *n* = 3), 48 h for ELISA of TGFβs proteins (D, *p < 0.05, **p < 0.01, *n* = 3), and 24 h and 72 h for immunostaining to pSMAD2/3 and α-SMA, respectively (E, nuclear staining by Hoechst 33342, Bar = 100 μm). Plastic and HA were used as controls

In the absence of TGFβ1, HCF and myofibroblasts cultured on immobilized HC-HA/PTX3 changed from spindle shape to cell aggregation without myofibroblast differentiation because TGFβ canonical signaling is suppressed as evidenced by the lack of nuclear translocation of pSMAD2/3 [35]. To determine whether such suppression was still maintained in the presence of TGFβ1, we repeated the above experiment by culturing passage 3 (P3) HCF on plastic with or without immobilized HA or HC-HA/PTX3 with or without TGFβ1. In agreement with our prior report [35], HCF cultured on HC-HA/PTX3, but not on plastic or HA, formed cellular aggregates within 24 h (h) and thereafter without TGFβ1 (Fig. 1A, up to Day 7). However, such cell aggregates were still maintained without nuclear pSMAD2/3 in 24 h (Fig. 1B and E) and cytoplasmic staining of α-SMA in 72 h (Fig. 1E) even with TGFβ1.

Without TGFβ1, expression of TGFβ1 mRNA and protein was significantly upregulated on plastic and HA but significantly downregulated in HCF cultured on HC-HA/PTX3 more so than with TGFβ1 (Fig. 1C and D, *p < 0.05, ^#^*p* < 0.05, downregulated, **p < 0.01, *n* = 3). Expression of TGFβ2 mRNA was significantly downregulated on plastic and HC-HA/PTX3 but not on HA (Fig. 1C, ^#^*p* < 0.05, downregulated, *n* = 3) while expression of TGFβ2 protein was not affected on either of these three substrates with TGFβ1 (Fig. 1D, all *p* > 0.05, *n* = 3). Interestingly, expression of TGFβ3 mRNA was significantly upregulated by all three substrates, especially by HC-HA/PTX3 (Fig. 1C, *p < 0.05, *n* = 3) while expression of TGFβ3 protein was significantly upregulated only by HC-HA/PTX3 (Fig. 1D, **p < 0.01, *n* = 3) with TGFβ1. These results collectively suggested that suppression of TGFβ canonical signaling was maintained only on immobilized HC-HA/PTX3 despite addition of TGFβ1 and accompanied by mitigation of TGFβ1 autoinduction and upregulation of TGFβ3.

**Fig. 2:**
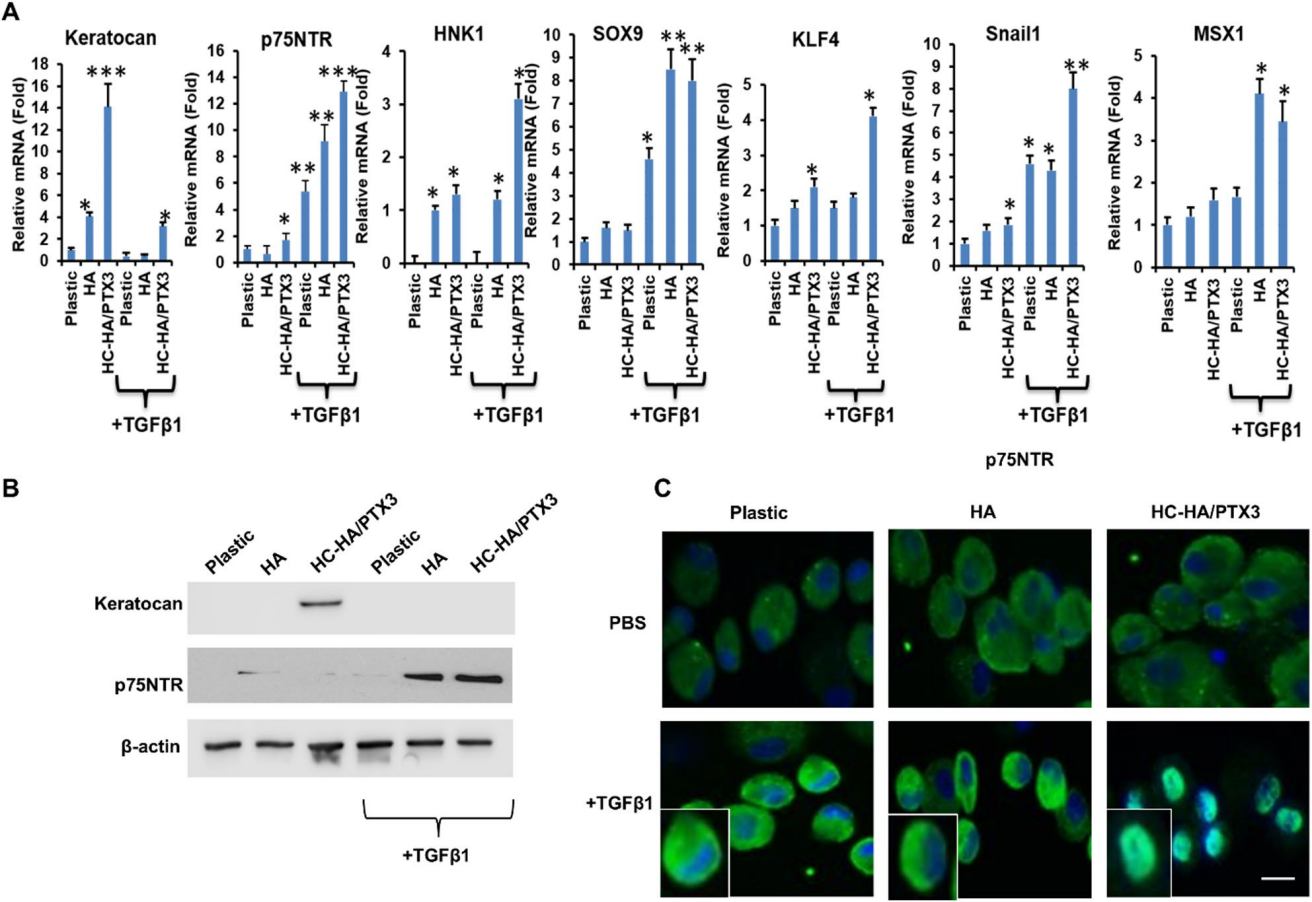
HC-HA/PTX3 downregulates keratocan but upregulates NC markers in the presence of TGFβ1. P3 HCF seeded on plastic with or without immobilized HA or HC-HA/PTX3 in DMEM + 10% FBS for 72 h were switched to DMEM + ITS for 24 h before being added with or without TGFβ1 for 24 h before qRT-PCR of keratocan and NC markers using the expression level on plastic set as 1 (**A**, *p < 0.05, **p < 0.01, ***p < 0.001, *n* = 3) and for immunostaining of p75NTR (**C**, nuclear staining by Hoechst 33342, Bar = 100 μm) as well as for 48 h before Western blot of keratocan and p75NTR proteins using β-actin as the loading control (**B**). Plastic and HA were used as controls.

Without TGFβ1, the resultant cellular phenotype on immobilized HC-HA/PTX3 was keratocyte as judged by upregulation of keratocan mRNA and protein (Fig. 2A, ***p < 0.001, *n* = 3) consistent with our prior report [35]. With TGFβ1, aforementioned upregulation of keratocan mRNA and protein by HCF on HC-HA/PTX3 was significantly reduced or aborted (Fig. 2A and B, *p < 0.05, *n* = 3), suggesting that these cells were no longer keratocytes. Compared to cultures without TGFβ1, the transcript expression of NC markers such as p75NTR, HNK1, Sox9, KLF4, Snail1, and MSX1 was particularly upregulated by HC-HA/PTX3 with TGFβ1 in comparison to HA or plastic (Fig. 2A, *p < 0.05, **p < 0.01, *n* = 3). Western blot confirmed that HC-HA/PTX3 uniquely upregulated keratocan without TGFβ1 as reported [35] and that such upregulation was aborted with TGFβ1 (Fig. 2B), suggesting the loss of keratocyte phenotype. In contrast, expression of p75NTR protein was upregulated from plastic to HA and HC-HA/PTX3 with TGFβ1 by 3.1- and 7.2-fold, respectively, suggesting the emergence of the NC phenotype (Fig. 2B, *p < 0.05 and **p < 0.01, *n* = 3). Intriguingly, immunostaining showed cytoplasmic staining of p75NTR protein in HCF cultured on plastic and HA but nuclear staining in cells cultured on HC-HA/PTX3 with TGFβ1 (Fig. 2C). These results collectively suggested that HCF cultured on immobilized HC-HA/PTX3 adopted a phenotype suggestive of NC progenitors in the presence of TGFβ1.

**Fig. 3:**
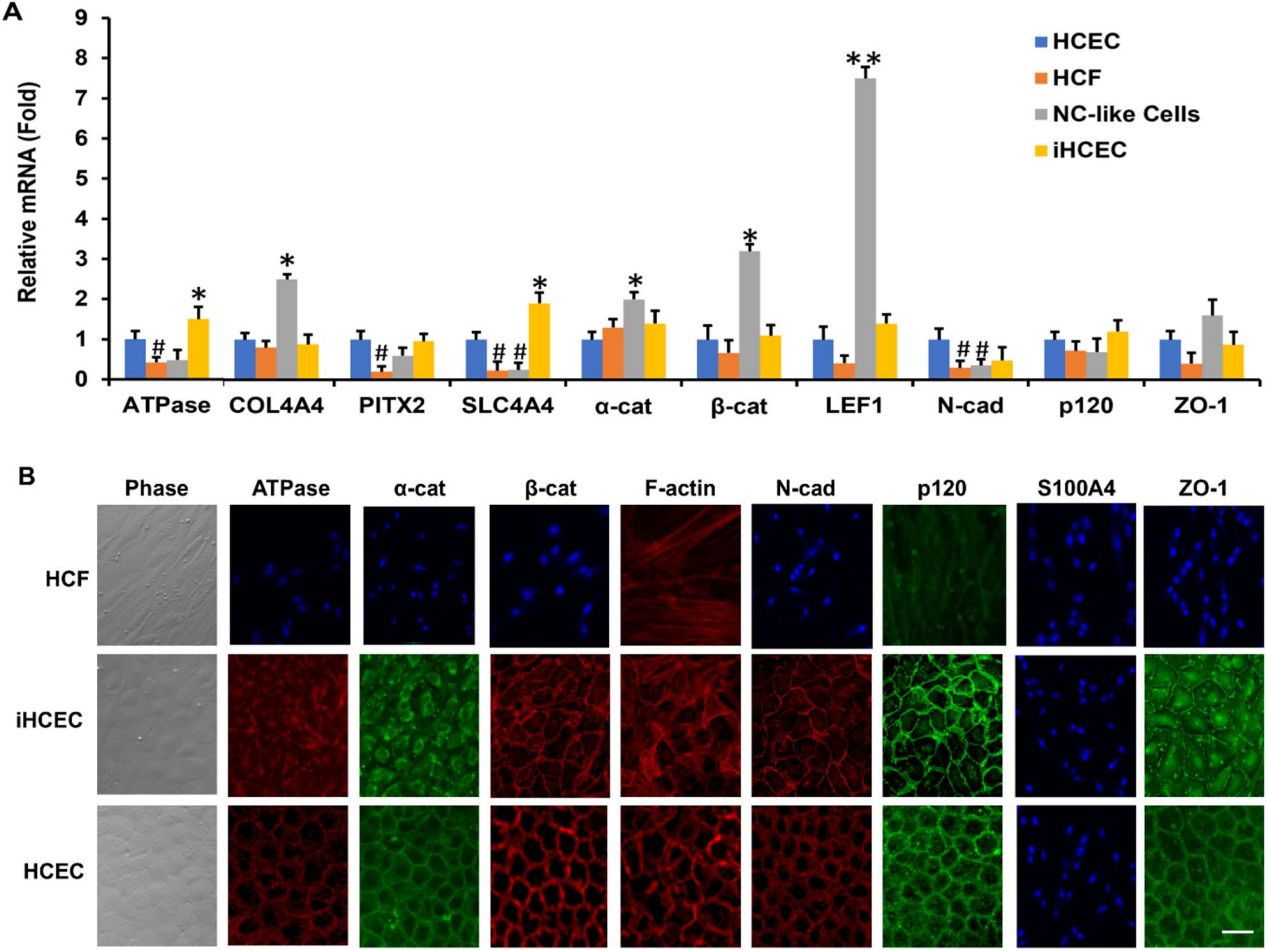
HC-HA/PTX3-reprogrammed NC cells can be induced into HCEC. NC cells generated from HCF after culture on immobilized HC-HA/PTX3 (see Legend in Fig. 2) were further cultured in low-glucose, low-calcium DMEM with 10% FBS for 3 weeks. Primary HCEC cultured on collagen IV coated dishes in MESCM plus 5% FBS and HCF cultured on plastic with TGFβ1 for 24 h were used as HCEC and HCF controls, respectively. HCEC markers were assessed by qRT-PCR using the expression level in HCEC set as 1 [**A**, *p < 0.05, **p < 0.01, ^#^*p* < 0.05, downregulated, *n* = 3] and by immunostaining (**B**, Bar = 100 μm)

To further confirm the reprogramming into NC progenitors, we cultured resultant cells after cultured on immobilized HC-HA/PTX3+TGFβ1 in DMEM + ITS for 24 h to see if they could differentiate into HCEC by switching the medium to low-glucose, low-calcium DMEM + 10% FBS for 3 weeks, a method used to induce human NC progenitors into HCEC [44]. Using the expression level by HCEC as the positive control and the expression level by HCF without immobilized HC-HA/PTX3+TGFβ1 as the negative control, transcript expression of HCEC markers such as Na-K-ATPase, COL4A4, PITX2, SLC4A4, β-catenin, LEF1, N-cadherin, p120 and ZO-1 in induced HCEC (iHCEC) was comparable to or higher than that of HCEC freshly isolated and cultured. In contrast, the transcript expression of COL4A4, α-catenin, β-catenin and LEF1 was upregulated while that of SLC4A4 and N-cadherin reduced in induced NC cells before HCEC induction. In addition, the transcript expression of Na-K-ATPase, PITX2, SLC4A4 and N-cadherin was downregulated in HCF before induction (Fig. 3A, *p < 0.05, ^#^*p* < 0.05, downregulated, **p < 0.01, *n* = 3). Immunostaining showed that iHCEC adopted hexagonal shape and cell junctional staining of Na-K-ATPase, α-catenin, β-catenin, F-actin, N-cadherin, p120 and ZO-1, not much different from HCEC (Fig. 3B) [46, 47]. Furthermore, expression of S100A4, a marker of endothelial to mesenchymal transition, was absent. These characteristics were not observed in NC cells before HCEC induction or HCF on plastic, which remained the fibroblastic shape (Fig. 3B). Collectively, these results confirmed that HCF was indeed reprogrammed into NC progenitors by HC-HA/PTX3 in the presence of TGFβ1.

### HC-HA/PTX3 upregulates cyclin D1 to downregulate TβRII and suppress TGFβ canonical signaling

**Fig. 4:**
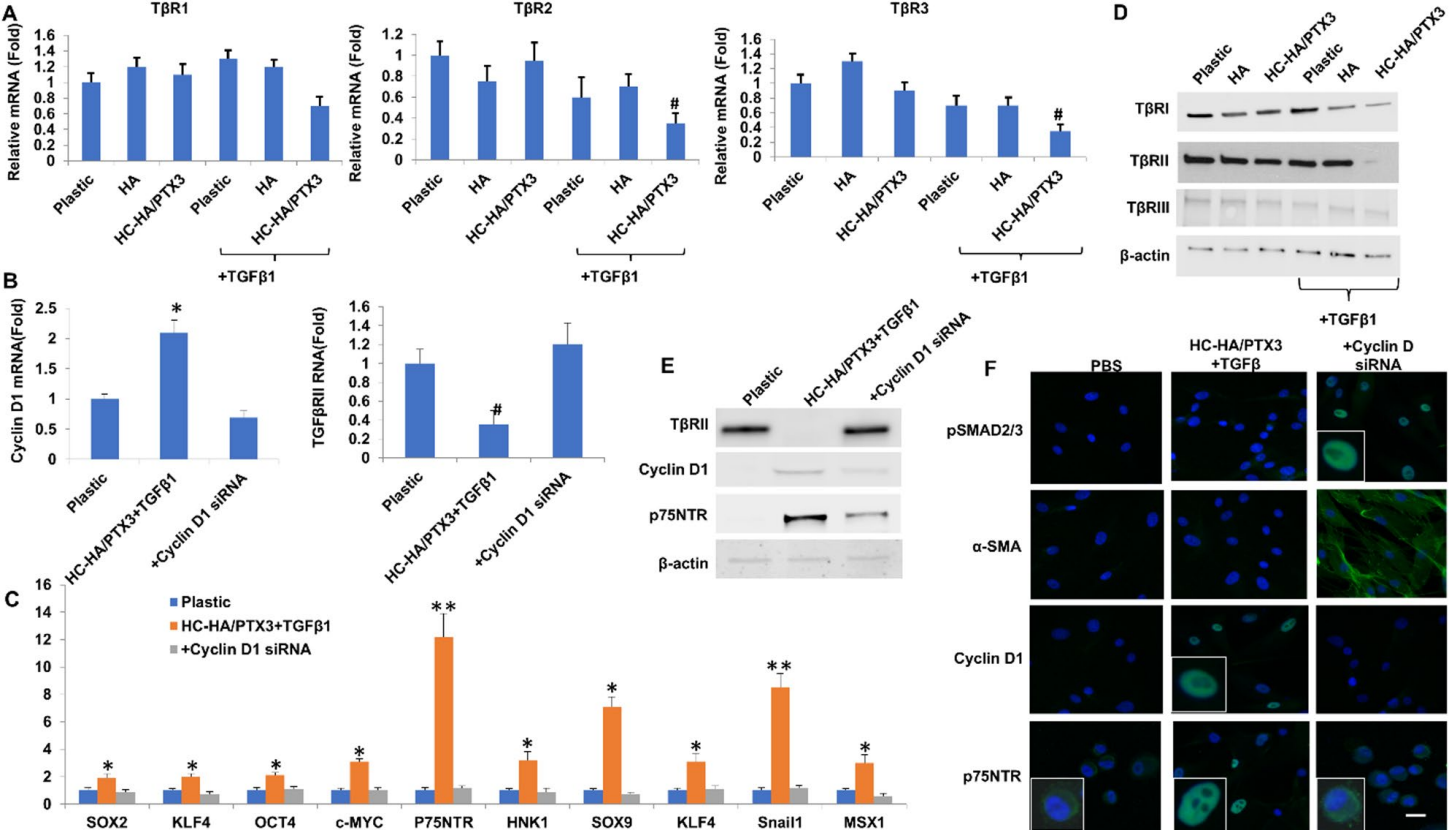
Downregulation of TβRII by upregulated cyclin D1 inhibits TGFβ canonical signaling during reprogramming. P3 HCF seeded on plastic with or without immobilized HA or HC-HA/PTX3 in DMEM + 10% FBS for 72 h were switched to DMEM + ITS for 24 h before being added with or without TGFβ1 with or without cyclin D1 siRNA for 24 h before qRT-PCR of TβRs, cyclin D1, ESC and NC markers using the expression level on plastic set as 1 (**A**–**C**, *^,#^*p* < 0.05, **p < 0.01, ***p < 0.001, *n* = 3) using plastic and HA as controls, for 48 h before western blot quantitation of TβRs, cyclin D1 and p75NTR using β-actin as the loading control (**D** and **E**), for 24 h before immunostaining of pSMAD2/3, cyclin D1 and p75NTR, and 72 h before immunostaining of α-SMA (**F**, nuclear staining by Hoechst 33342, Bar = 100 μm) using PBS as the control

Because HCF cultured on immobilized HC-HA/PTX3 withstood the challenge of exogenous TGFβ1 as evidenced by suppression of TGFβ canonical signaling (Fig. 1), we investigated whether there might be a change in expression of TβRs. Without TGFβ1, expression of TβRs mRNA and protein was not altered when cultured on plastic, HA or HC-HA/PTX3 (Fig. 4A, *p* > 0.05, *n* = 3). In contrast, expression of TβRII and TβRIII mRNA was reduced by 3-fold and 4-fold, respectively, when cultured on HC-HA/PTX3 with TGFβ1 (Fig. 4A, ^#^*p* < 0.05, downregulated, *n* = 3). Surprisingly, TβRII protein was uniquely suppressed to nil when cultured on HC-HA/PTX3 with TGFβ1 (Fig. 4D, #*p* < 0.05, *n* = 3). These results indicated that suppression of TGFβ canonical signaling was associated with marked downregulation of TβRII but not TβRI or TβRIII when cultured on HC-HA/PTX3 with TGFβ1.

Because the TβRII promoter contains an inverted CCAAT box [48], cyclin D1 inhibits CCAAT box-containing enhancer binding proteins (c/EBP) [49], and overexpression of cyclin D1 downregulates TβRII but not TβRI [51, 52], we assessed if cyclin D1 was also upregulated by HC-HA/PTX3 to account for downregulating TβRII mRNA and protein. Upregulation of cyclin D1 mRNA by 2.0-fold and cyclin D1 protein by 4.1-fold was noted in HCF cultured on HC-HA/PTX3 with TGFβ1 (Fig. 4B and E, *p < 0.05, *n* = 3). Furthermore, such upregulation of cyclin D1 mRNA and protein was accompanied by nuclear staining of cyclin D1 (Fig. 4F). Upregulation of cyclin D1 was correlated with downregulation of TβRII mRNA (Fig. 4B) and protein (Fig. 4D). Knockdown by cyclin D1 siRNA that downregulated expression of cyclin D1 mRNA and protein and nuclear localization led to upregulation of TβRII mRNA and protein (Fig. 4B and D), aborted transcript overexpression of ESC and NC markers (Fig. 4C, all *p* > 0.05, *n* = 3), downregulated expression of p75NTR protein by 3.1-fold (Fig. 4E, *p* < 0.05, *n* = 3), inhibited nuclear staining of p75NTR (Fig. 4F), and restored nuclear staining of pSMAD2/3 and cytoplasmic staining of α-SMA (Fig. 4F). In addition, “HC-HA/PTX3 upregulated mRNA expression of SOX2, KLF4, OCT4, and c-MYC in 24 h by approximately 2-, 2-, 2- and 3-times, respectively. Such an upregulated was inhibited by cyclin D1 siRNA. These results collectively suggested that cyclin D1 was upregulated by HC-HA/PTX3 in the presence of TGFβ1 to downregulate TβRII. TβRII downregulation by knockdown of cyclin D1 was causally linked to the loss of reprogramming into the NC phenotype and loss of expression of ESC markers, and activation of TGFβ canonical signaling leading to myofibroblasts.

### Binding of HC-HA/PTX3 with CD44 is required to sequestrate TβRII in lipid raft

**Fig. 5:**
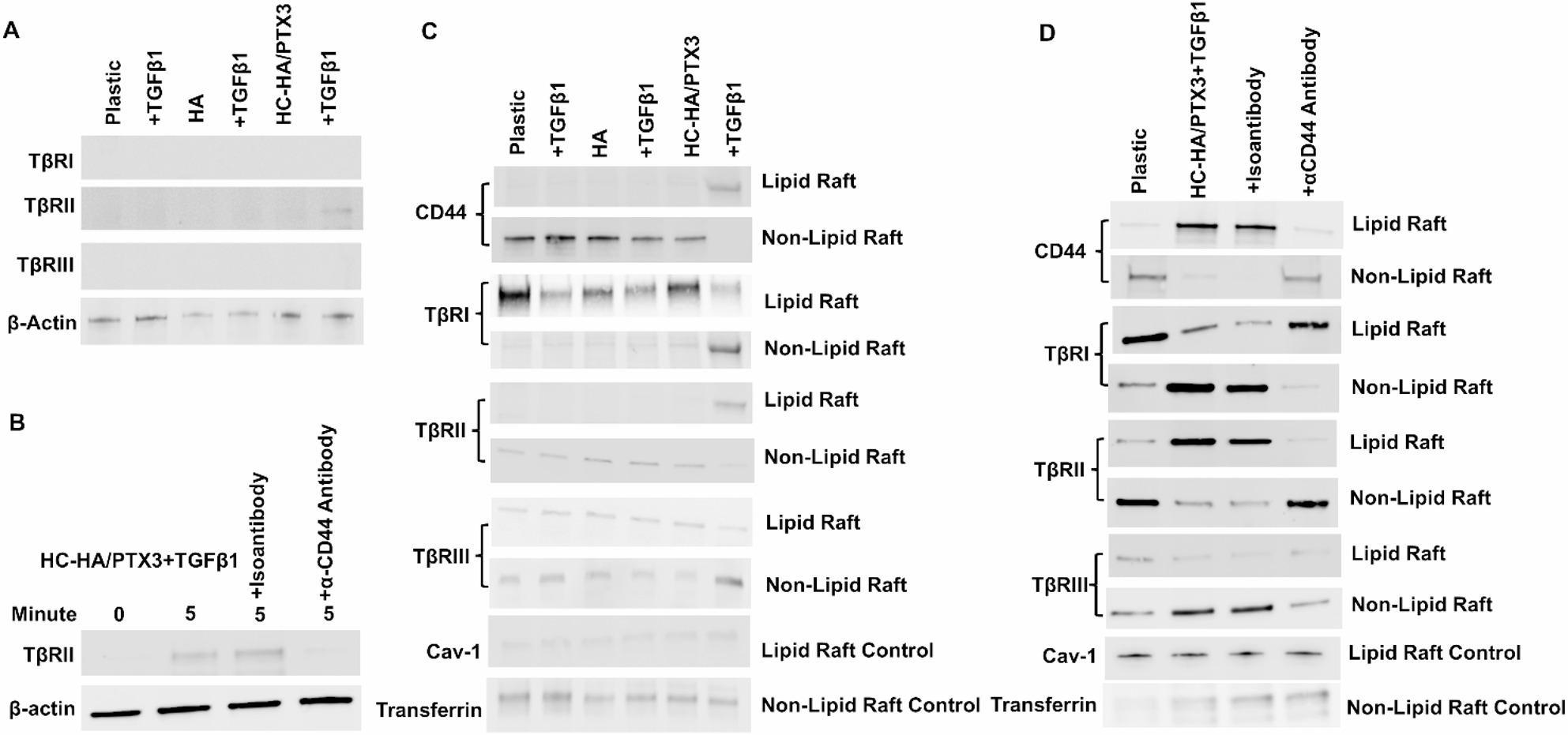
Binding of HC-HA/PTX3 with CD44 sequestrates TβRII and CD44 in lipid raft. P3 HCF seeded on plastic in DMEM + 10% FBS were switched to DMEM + ITS for 24 h before being treated with PBS, soluble HA or HC-HA/PTX3 with or without TGFβ1 and with or without addition of α-CD44 antibody or isoantibody for 5 min before the membrane and cytoplasmic preparation was precipitated with CD44 antibody and Western blot of TβRs using β-actin as the loading control (**A**, **B**). The above cultures were also harvested for lipid raft and non-lipid raft separation before Western blot using antibodies against CD44 and TβRs while using caveolin-1 and transferrin as lipid and non-lipid raft controls, respectively (**C**, **D**). Plastic and HA were used as controls

Because our previous studies have demonstrated that soluble HC-HA/PTX3 is comparable to immobilized HC-HA/PTX3 in causing cell aggregation, mRNA expression of SDF1, CXCR4, BMPs and NC markers in human limbal niche cells [35, 36], we changed to soluble HC-HA/PTX3 for the ease of the remaining experiments. Because CD44 can physically bind to TβRI or TβRII as a complex [53, 54] and is a receptor responsible for binding with HA and HC-HA/PTX3 as reported [55], we performed immunoprecipitation with an anti-CD44 antibody followed by Western blot with respective anti-TβRI-III antibodies using total membrane and cytoplasmic preparation of HCF 5 min after cultured with soluble HA or HC-HA/PTX3 with or without TGFβ1. The results showed that CD44 was coimmunoprecipitated with TβRII only when cells were cultured in HC-HA/PTX3 with TGFβ1 (Fig. 5A) and that such binding between CD44 and TβRII was blocked by an α-CD44 antibody (Fig. 5B). These findings suggested that binding between CD44 and HC-HA/PTX3 was required to facilitate CD44’s binding to TβRII in the presence of TGFβ1. To discern whether the above event took place in lipid or non-lipid rafts, we prepared both rafts from HCF cultured on plastic with or without HA or HC-HA/PTX3 5 min after treatment with or without TGFβ1 and performed western blot using antibodies against CD44 and each TβR. Using caveolin and transferrin as a marker for lipid and non-lipid raft, respectively, we noted that both CD44 and TβRII were exclusively moved from non-lipid raft to lipid raft only when cells were cultured in HC-HA/PTX3 with TGFβ1 (Fig. 5C). On the contrary, TβRI and TβRIII predominantly moved from lipid raft to the non-lipid raft (Fig. 5C). Selective sequestration of TβRII with CD44 in lipid raft was also blocked by an α-CD44 antibody (Fig. 5D), suggesting that binding of CD44 with HC-HA/PTX3 was necessary to elicit subsequent CD44’s binding and sequestration of TβRII in lipid raft in the presence of TGFβ1.

### Activation of TGFβ noncanonical signaling via TAK1-cJUN

**Fig. 6:**
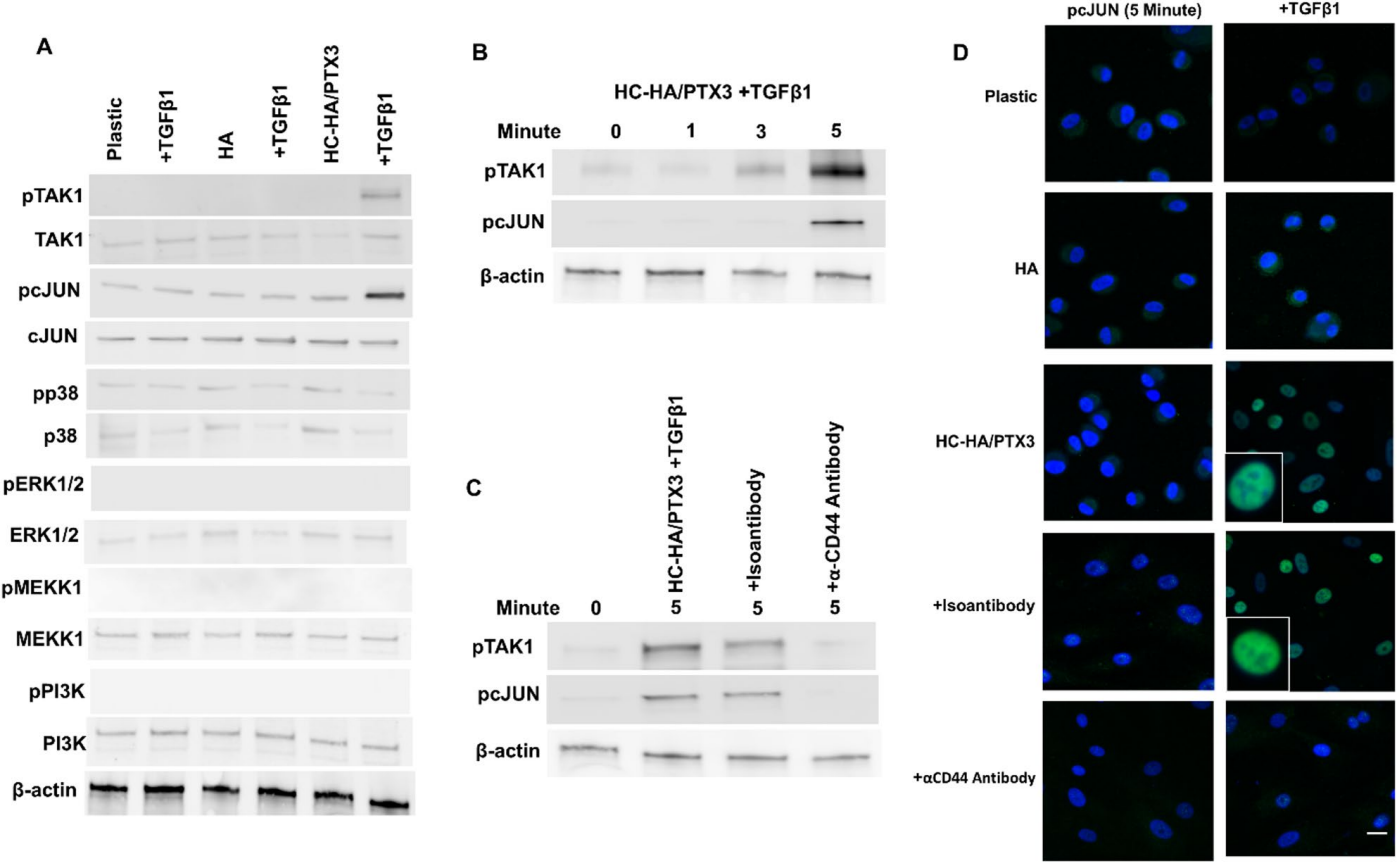
Activation of TAK1-cJUN signaling with nuclear pcJUN is abolished by α-CD44 antibody. P3 HCF seeded on plastic in DMEM + 10% FBS for 72 h were switched to DMEM + ITS for 24 h before being treated with or without soluble HA or HC-HA/PTX3 and with or without exogenous TGFβ1 for 5 min. Total membrane and cytoplasmic extracts were prepared for Western blot of various phospho-kinases using β-actin as the loading control (**A**). The same experiment was repeated for a time course up to 5 min for Western blot of pTAK1 and pcJUN using β-actin as the loading control (**B**). The above cultures were also pre-incubated with α-CD44 antibody or isoantibody for 30 min followed by addition of soluble HC-HA/PTX3 and TGFβ1 for 5 min before Western blot of pTAK1 and pcJUN using β-actin as the loading control (**C**) and immunostaining of pcJUN (**D**, bar = 100 μm). Plastic and HA were used as controls

Although sequestering of TβRII by CD44 in lipid raft after binding of HC-HA/PTX3 with CD44 supported suppression of TGFβ1 canonical signaling, TβRI and TβRIII in non-lipid raft prompted us to examine whether TGFβ noncanonical (SMAD-independent) signaling was activated. As a first step, we used Western blot to screen various phospho-kinases that might be involved in TGFβ noncanonical signaling. Among all surveyed, phosphorylation of TAK1 (pTAK1) and cJUN (pcJUN) was uniquely upregulated 5 min after HCF were cultured with HC-HA/PTX3 only when TGFβ1 was added (Fig. 6A). pTAK1 was promoted by 2-fold as early as 3 min and increased to 4.5-fold by 5 min while pcJUN was undetectable at baseline but became highly detectable by 5 min (Fig. 6B, all *p* < 0.05, *n* = 3). Importantly, such upregulation of pTAK1 and pcJUN was abolished when cells were pre-incubated with an α-CD44 antibody for 30 min but not an isotype antibody (Fig. 6C). Immunostaining confirmed nuclear staining of pcJUN as early as 5 min in cells cultured with soluble HC-HA/PTX3, but not with soluble HA or plastic, only in the presence of TGFβ1 (Fig. 6D). Furthermore, such nuclear staining of pcJUN was abolished by an α-CD44 antibody (Fig. 6D), suggesting that activation of TAK1-cJUN signaling by HC-HA/PTX3 in the presence of TGFβ1 also required binding with CD44.

### Activation of TAK1-cJUN signaling leads to nuclear staining of cyclin D1 and expression of NC markers

**Fig. 7:**
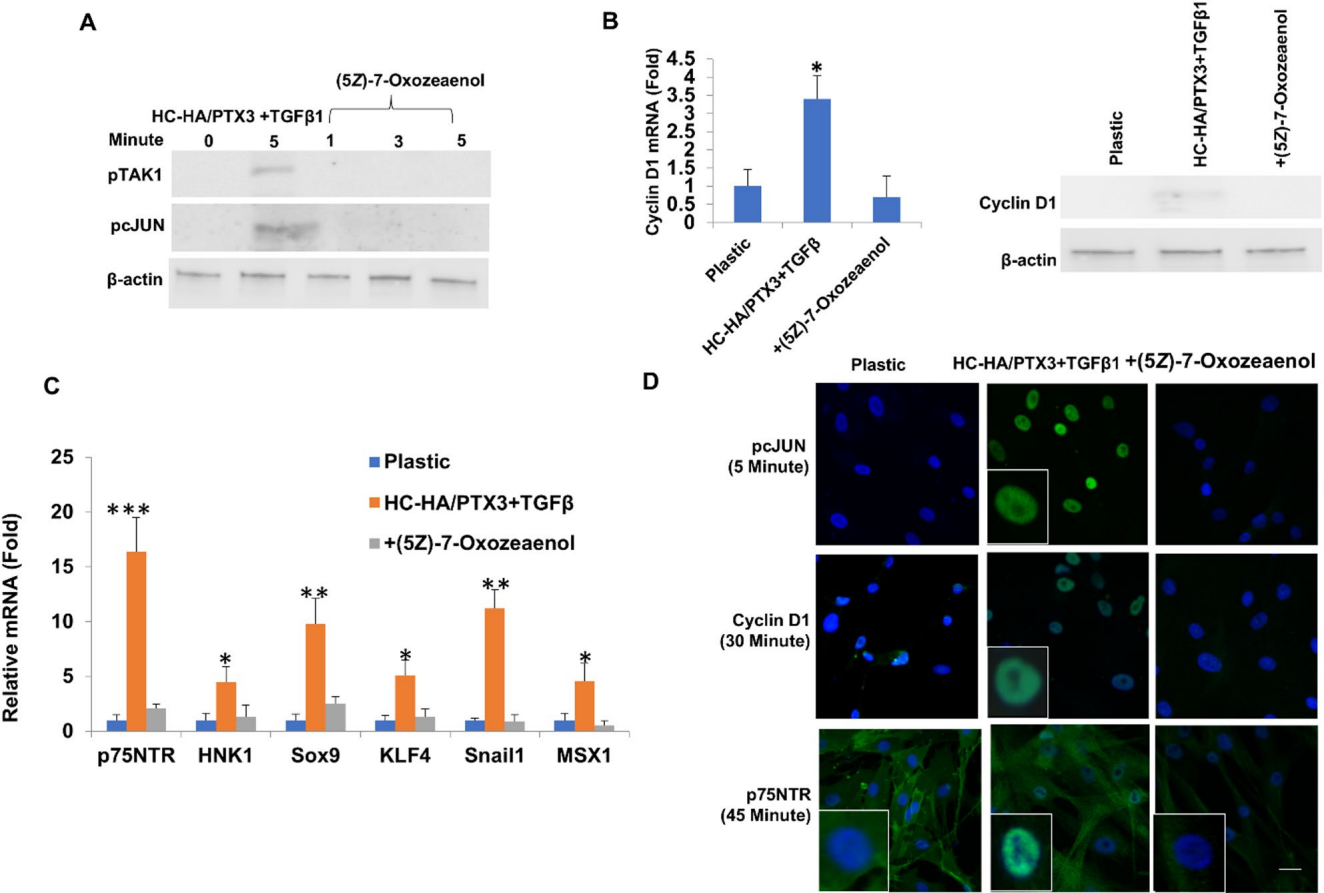
Inhibition of TAK1 by (5*Z*)-7-Oxozeaenol abolishes nuclear cyclin D1 and p75NTR. P3 HCF seeded in DMEM + 10%FBS on plastic for 72 h were switched to DMEM + ITS for 24 h before being added with HC-HA/PTX3+TGFβ1 with or without (5*Z*)-7-Oxozeaenol up to 5 min before Western blot of pTAK1 and pcJUN using β-actin as the loading control (**A**), for 48 h before Western blot of cyclin D1 using β-actin as the loading control (**B**), and for 24 h before qRT-PCR of transcripts of cyclin D1 and NC markers using the expression level on plastic set as 1 (**B** and **C**, *p < 0.05, **p < 0.01 and ***p < 0.001, *n* = 3). P3 HCF cultured on plastic with or without HC-HA/PTX3+TGFβ1 for 0, 5, 30 and 45 min before immunostaining of pcJUN, cyclin D1, or p75NTR (**D**, Bar = 100 μm). Plastic was used as the control

We then examined whether activation of TAK1-cJUN signaling led to upregulation of cyclin D1 which downregulated TβRII and suppressed TGFβ canonical signaling (Fig. 4) by pre-treatment with (5*Z*)-7-oxozeaenol, a specific TAK inhibitor [56]. The results showed that such pre-treatment completely inhibited phosphorylation of TAK1 and cJUN (Fig. 7A) and downregulated cyclin D1 mRNA and protein (Fig. 7B). In addition, nuclear staining of pcJUN in 5 min, cyclin D1 in 30 min and p75NTR in 45 min were also abolished by the pretreatment (Fig. 7D). Furthermore, the above findings were accompanied by downregulation of cyclin D1 mRNA and protein and NC marker mRNA (Fig. 7B and C, ^*^p < 0.05, **p < 0.01 and ***p < 0.001, *n* = 3). Therefore, the activation of TAK1-cJUN signaling was causally associated with upregulation of cyclin D1 and expression of NC genes.

### Activation of CD44ICD signaling also leads to nuclear staining of cyclin D1 and p75NTR

**Fig. 8:**
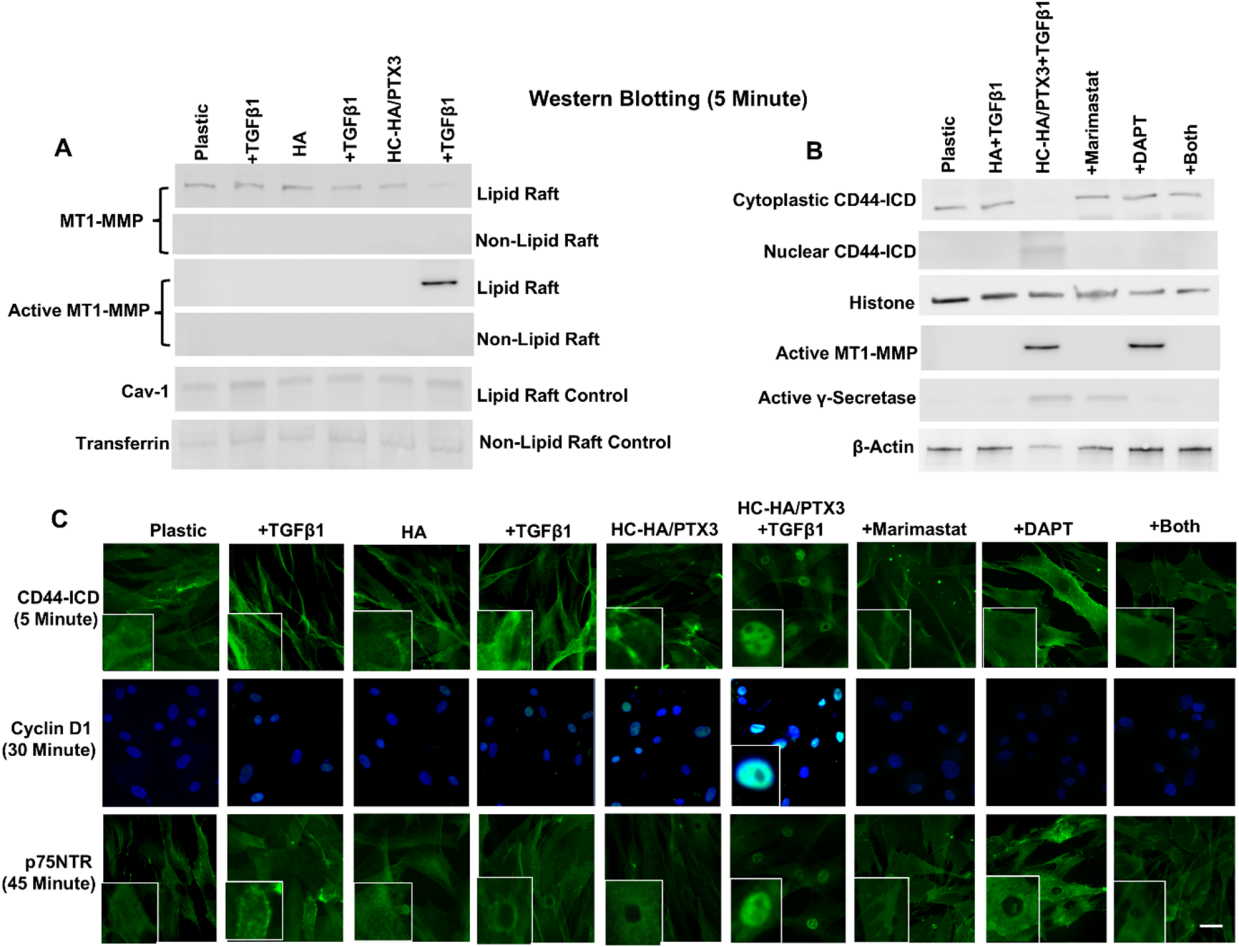
Activation of CD44ICD signaling also leads to nuclear cyclin D1 and p75NTR. P3 HCF seeded on plastic in DMEM + 10% FBS for 24 h and DMEM + ITS for 24 h before being treated with or without soluble HA or HC-HA/PTX3 with or without TGFβ1 and with or without pretreatment of Marimastat or DAPT. Lipid and non-lipid raft preparation (**A**, using caveolin-1 and transferrin as respective controls) or cytoplasmic and nuclear preparation (**B**, using b-actin and histone as respective controls) were subjected to Western blot using antibodies against MT1MMP and catalytic domain of MT1MMP (active MT1MMP) (**A**) or an antibody against CD44ICD (**B**). The above cell cultures at 5, 15, 30 and 45 min after the treatment were also subjected to immunostaining of CD44ICD, cyclin D1 and p75NTR (C, bar = 100 μm). Plastic and HA were used as controls

Because CD44ICD released by cleavage of the ectoplasmic domain by MT1MMP followed by cleavage of intracytoplasmic domain by γ-secretase [57, 58] can elicit signaling by nuclear translocation to transactivate genes [59], we examined whether CD44ICD was also released to transactive cyclin D1. In HCF cultured in HC-HA/PTX3 5 min after addition of TGFβ1, active MT1MMP was found in lipid raft while inactive MT1MMP was absent (Fig. 8A). In contrast, inactive MT1MMP was found in lipid raft without any active MT1MMP for all other treatment conditions (Fig. 8A). This result indicated unique activation of MT1MMP in cultures with HC-HA/PTX3 after TGFβ1 treatment. To prove whether CD44ICD might be transferred to the nucleus, we pretreated cultures for 30 min with Marimastat, a broad-spectrum inhibitor of MMPs including MT1MMP, with or without DAPT, a specific γ-secretase inhibitor before cytoplasmic and nuclear preparations for Western blot analysis. The result showed nuclear localization of CD44ICD (Fig. 8B) together with nuclear staining of CD44ICD (Fig. 8C) as early as 5 min only in cells treated with HC-HA/PTX3+TGFβ1. Interruption of CD44ICD formation by Marimastat, DAPT, or both abolished nuclear staining of cyclin D1 at 30 min (Fig. 8C) and nuclear staining of p75NTR at 45 min (Fig. 8C) in cultures with HC-HA/PTX3 with TGFβ1. Collectively, these results suggested that nuclear CD44ICD signaling was also elicited by HC-HA/PTX3 with TGFβ1 resulting in subsequent nuclear staining of cyclin D1 and nuclear staining of p75NTR.

## Discussion

### Main findings

**Fig. 9:**
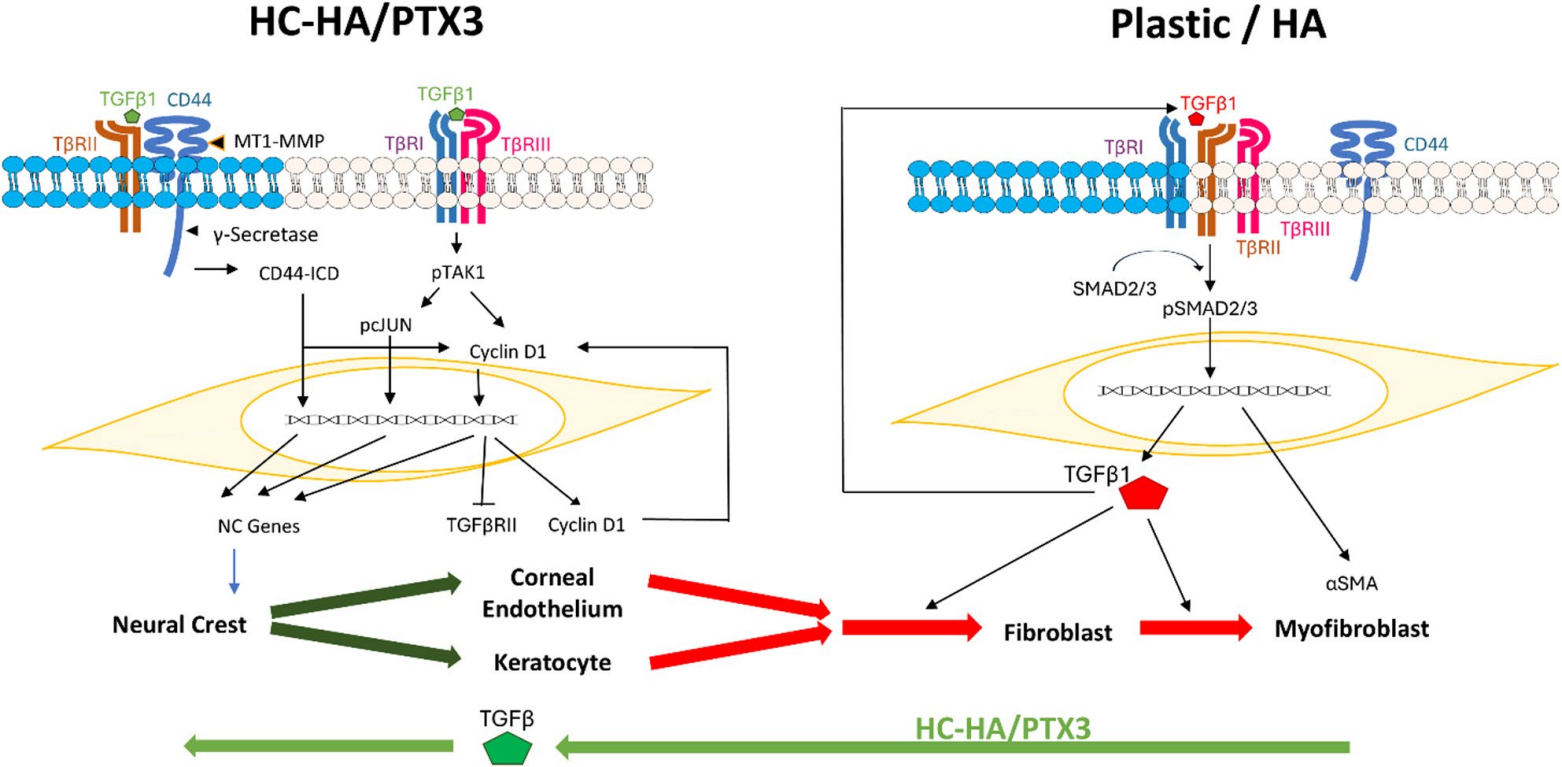
Reprogramming HCF to NC progenitors by HX-HA/PTX3 in the presence of TGFβ1 HC-HA/PTX3 with TGFβ1 reprograms HCF into NC progenitors by preventing myofibroblast differentiation and reprograming NC progenitors. HC-HA/PTX3’s reprogramming of HCF to NC progenitors requires suppression of TGFβ canonical signaling starting from sequestration of TβRII in lipid raft upon binding with CD44 and ending with downregulation of TβRII by nuclear cyclin D1. HC-HA/PTX3 reprogramming further requires activation of noncanonical TAK1-cJUN via TGFβ1 binding to TβRI and nuclear translocation of CD44ICD which also leads nuclear cyclin D1. The sequential activation cascade of cyclin D1 by HC-HA/PTX3-TGFβ1→TAK1→cJUN and HC-HA/PTX3-CD44→CD44ICD drives such reprogramming.  stands for Lipid Raft.  stands for Non-lipid Raft

HC-HA/PTX3 from human amniotic membrane may not only prevent human corneal fibroblasts from differentiation into myofibroblasts by suppressing TGFβ canonical signaling but also reprogram into neural crest progenitors by switching to noncanonical TAK1/cJun signaling and nuclear CD44ICD signaling. This signaling paradigm unravels the regenerative potential of HC-HA/PTX3 to reverse scar even in the presence of pro-fibrotic TGFβ1 (see Fig. 9).

### Interpretation and comparison with literature

TGFβ’s profibrotic effect is mediated by canonical SMAD signaling by promoting scar formation including differentiation of αSMA-expressing myofibroblasts [17–20]. This notion is recapitulated in HCF cultured on plastic or HA where myofibroblast differentiation in the presence of TGFβ1 is mediated by activation of TGFβ canonical signaling as evidenced by nuclear staining of pSMAD2/3 (Fig. 1). Our study further suggests that activation of TGFβ canonical signaling under these two culturing conditions is augmented by autoinduction of TGFβ1 (summarized in Fig. 9). In contrast, when HCF are cultured on HC-HA/PTX3, TGFβ canonical signaling is suppressed not only in the absence of TGFβ1 [35] but also in the presence of TGFβ1 (Fig. 1). Suppression of TGFβ canonical signaling is further aided by downregulation of TGFβ1 autoinduction and upregulation of anti-scaring TGFβ3. Collectively, the paradigm summarized in Fig. 9 reiterates that HC-HA/PTX3 is the prime candidate responsible for AM’s anti-scarring action by suppressing TGFβ canonical signaling to prevent myofibroblasts differentiation.

Activation of TGFβ canonical signaling requires heterodimerization of TβRII with TβRI [1, 2]. Suppression of TGFβ canonical signaling by HC-HA/PTX3 starts soon (in 5 min) after HC-HA/PTX3 binds to CD44 to sequester TβRII as evidenced by coimmunoprecipitation with TβRII exclusively in lipid raft while leaving TβRI and TβRIII predominantly in non-lipid raft (Fig. 5). As summarized in Fig. 9, HC-HA/PTX3 suppression of TGFβ canonical signaling ends with marked downregulation of TβRII in 48 h, which is causally linked to nuclear translocation of cyclin D1 (Fig. 4). Because TβRI and TβRIII are left in non-lipid raft after sequestration of TβRII by CD44 in lipid raft (Fig. 5), TGFβ1 binding with TβRI activates TGFβ noncanonical signaling via TAK1-cJUN, which leads to nuclear cyclin D1(Fig. 7). Similar activation of TAK1 signaling is observed in palatal mesenchyme cells of TβRII knockout mice where TβRI and TβIII are operating without TβRII [60]. Nuclear translocation of cyclin D1 is also promoted by nuclear translocation of CD44ICD after activation of MT1MMP in lipid raft followed by γ-secretase cleavage (Fig. 8).

Suppression of TGFβ canonical signaling is a prerequisite to reprogram human dermal fibroblasts to osteoblasts [61] and to reprogram partially reprogrammed to fully reprogrammed induced pluripotent stem cells (iPSCs) by bypassing the requirement of Nanog [62, 63]. Herein, we also note that suppression of TGFβ canonical signaling by HC-HA/PTX3 is required to reprogram HCF to NC progenitors, of which the phenotype is suggested by expression of NC genes including overexpression and nuclear translocation of p75NTR (Fig. 2), a marker for retinal stem cells [64] and confirmed by successive induction into HCEC-like cells (Fig. 3).

However, suppression of TGFβ canonical signaling is not sufficient and requires activation of other signaling to achieve reprogramming of HCF. In the absence of TGFβ1, reprogramming into keratocytes by HC-HA/PTX3 (Fig. 2A and B) requires activation of nuclear translocation of CXCR4 followed by activation of BMP canonical signaling [35]. In the presence of TGFβ1, reprogramming into NC progenitors by HC-HA/PTX3 requires sequential activation cascade of cyclin D1 by HC-HA/PTX3-TGFβ1→TAK1→cJUN and HC-HA/PTX3-CD44→CD44ICD because nuclear translocation of both cJUN and CD44-ICD occurs as early as 5 min, while that of cyclin D1 occurs much later at 30 min (Figs. 7 and 8, respectively). Overexpression of cyclin D1 may not only suppress expression of TβRII gene but also help reprogramming as overexpression of cyclin D1 reprograms differentiated human epidermal cells into stem cell-like cells [65] and that cyclin D1 knockout in mouse embryonic fibroblasts blocks mesenchymal to epithelial transition (MET) required for initiating reprogramming [66].

The role of nuclear cJUN in promoting regeneration has been observed in Schwann cells as a downstream molecule of TAK1 to reprogram injured nerves to progenitors toward regeneration [67]. Nuclear pcJUN transactivates the OCT4 promoter to maintain stemness in HepG2-iPS-like cells [68]. Our finding of nuclear CD44ICD triggered by HC-HA/PTX3 in the presence of TGFβ1 in HCF is unique as nuclear CD44ICD has been exclusively shown in cancer cells to activate stemness factors such as Nanog, Sox2 and Oct4 [69] and to reprogram cancer stem cell properties [70]. Similarly, HC-HA/PTX3 upregulated mRNA expression of SOX2, KLF4, OCT4, and c-MYC in 24 h by approximately 2-, 2-, 2- and 3-times, respectively. Such an upregulated was also inhibited by cyclin D1 siRNA (Fig. 4C). Therefore, reprogramming of HCF to NC progenitors by HC-HA/PTX3 mimics that of classical stem cell pathways leading to iPSCs in morphological changes of mesenchymal epithelial transition, i.e., to form cell aggregation by spindle fibroblasts and upregulation of SOX2, KLF4, OCT4, and c-MYC transcripts. These findings allow us to surmise that similar reprogramming to classical stem cell pathways might be undertaken by HC-HA/PTX3.

HC-HA/PTX3 may exert not only anti-inflammatory actions by modulating both innate and adaptive immune responses [37, 41] but also a direct anti-scarring action as reported [33–36, 70]. These anti-inflammatory and anti-scarring properties plus the finding that HC-HA/PTX3 has also been reported to reprogram aging human limbal niche cells that have lost nuclear Pax6 expression after sequential passaging to their in vivo progenitor phenotype with nuclear Pax6 expression and restoring their ability of supporting limbal epithelial SC renewal and quiescence [33] as well as reprogramming potential presented herein suggest that HC-HA/PTX3 might help address corneal fibrosis involving corneal fibroblasts but also fibrosis caused by other mesenchymal cell types, such as dermal, cardiac, or pulmonary fibroblasts. Indeed, subconjunctival injection of HC-HA/PTX3 prolongs the graft survival in a murine model of allograft corneal transplantation [70]. Subconjunctival lacrimal injection of HC-HA/PTX3 prevents scarring of conjunctiva and lacrimal glands, thus avoiding loss of goblet cells and preserving tear production in a murine model of graft-versus-host diseases [71]. Cryopreserved amniotic membrane containing HC-HA/PTX3 is being evaluated in patients with neurotrophic keratitis and dry eye disease. Future in vivo investigation of HC-HA/PTX3-containing biologics will be directed to treating corneal scar or reversal of scar formation in the trabecular meshwork of glaucomatous eyes.

Additionally, recent literature further supports the notion that HC-HA/PTX3 containing amniotic membrane-derived or umbilical cord-derived bioactive complexes [70, 72–76] exert similar regenerative and antifibrotic benefits [35, 37].

### Strengths and limitations

*Strengths* We discovered for the first time that HC-HA/PTX3 might (1) prevent HCF to differentiate into myofibroblasts due to suppression of TGFβ canonical signaling, (2) reprogram HCF into NC progenitors by switching TGFβ canonical to noncanonical signaling that involves TAK1/cJun, TAK1-nuclear cyclin D1 and CD44ICD, (3) engineer “autologous” graft for tissue replacement.

*Limitations* Although in HEK293T cancer cells, the CD44 intracellular domain (ICD) interacts with the transcription factor cAMP-response element binding protein (CREB) to promote its phosphorylation, binding, and transcriptional activity of the cyclin D1 gene [77], We do not have direct experimental evidence, e.g., CHIP or promoter-reporter assays, to resolve whether this was the case for nuclear CD44ICD to promote transcript expression and nuclear transactivation of cyclin D1 in HCF. This mechanistic limitation requires further investigation. For iPSCs, cellular aggregation highlighting mesenchymal to epithelial transition (MET) is an early event leading to reprogramming [78]. In our model system, we also note cellular aggregation in HCF following exposure to HC-HA/PTX3 before embarking on reprogramming in the absence of TGFβ1 [35] and in the presence of TGFβ1 (Fig. 1). Future studies are needed to determine how cellular aggregation of HCF that requires nuclear transfer of CXCR4 occurs and withstands the challenge of TGFβ1 upon exposure to HC-HA/PTX3 (Fig. 1).

## Conclusions

The action paradigm summarized in Fig. 9 shows how HC-HA/PTX3 from AM may not only prevent HCF from differentiation into myofibroblasts by suppressing TGFβ canonical signaling but also reprogram HCF into NC progenitors by switching to TGFβ noncanonical signaling that involves TAK1/cJun, TAK1-nuclear cyclin D1 and CD44ICD. Therefore, when fibroblasts are exposed to HC-HA/PTX3 as an ECM, the traditional pro-fibrotic role of TGFβ1 (Fig. 9, marked in red) can be turned into a pro-regenerative role (marked in green). The finding that NC progenitors generated by HC-HA/PTX3 reprogramming of HCF can be induced into HCEC (Fig. 9, green arrows) also suggests that tissue engineering of “autologous” graft for tissue replacement is feasible. In conclusion, this signaling paradigm unravels the regenerative potential of HC-HA/PTX3 to reverse scar toward regeneration even in the presence of pro-fibrotic TGFβ1.

## Supplementary Information

Below is the link to the electronic supplementary material.

Supplementary Material 1.

## Data Availability

Available upon request.
