# Supplementary material for: HC-HA/PTX3 from amniotic membrane reprograms human corneal fibroblasts to neural crest progenitors by switching from canonical to noncanonical TGFβ signaling
Source: Stem Cell Res Ther. 2026 Mar 21;17:162. doi: 10.1186/s13287-026-04983-w (PMC13126780; doi:10.1186/s13287-026-04983-w)
Supplement: Supplementary file 1 — Supplementary Material 1. [file 13287_2026_4983_MOESM1_ESM.docx]

**Supplemental Data**

**Table S1.** Material Used for Cell Isolation, Expansion and Experiments.

| **Name** | **Source** | **Concentration** |
| --- | --- | --- |
| 1-ethyl-3(3-dimethylaminopropyl) carbodiimide (EDAC) | Thermo Fisher Scientific, Indianapolis, IN | 6.15 mg/mL |
|  |  |  |
| (5Z)-7-oxozeaenol | Sigma-Aldrich, St Louis, MO | 20 nM |
| 0.05%Trypsin-EDTA (T/E) | Invitrogen, Grand Island, NY | 0.25% and 1mM |
| Amphotericin B | Invitrogen, Grand Island, NY | 50µg/mL |
| Bovine serum albumin (BSA) | Sigma-Aldrich, St Louis, MO | 40 mg/mL |
| Collagenase A | Roche, Indianapolis, IN | 2 mg/mL |
| Covalink-NH 96 wells | Thermo-Fisher Scientific, Waltham, MA |  |
| Cyclin D1 siRNA | Thermo-Fisher Scientific, Waltham, MA | 100 nM |
| DMEM | Invitrogen, Grand Island, NY | DMEM/F-12 (1:1) |
| Dimethyl Sulfoxide | Sigma-Aldrich, St. Louis, MO | 0.1% |
| CsCl | Sigma-Aldrich, St. Louis, MO | gradient |
| Dispase | ThermoFisher Scientific, Waltham, MA | 10 mg/ml |
| DTT | Sigma-Aldrich, St. Louis, MO | 100 mM |
| Fetal Bovine Serum | Atlas, Fort Collins, CO. | 100% Bovine Serum |
| Gentamicin | Invitrogen, Grand Island, NY | 1.25 µg/mL |
| High Capacity Reverse Transcription Kit | Thermo-Fisher Scientific, 4368813, Waltham, MA |  |
| RNeasy Micro Kit | Qiagen, 74004, Valencia, CA |  |
| Sodium hyaluronate (HA) | Abbott, Santa Ana, CA | 20 µg/mL |
| Hyaluronidase | Sigma-Aldrich, St Louis, MO | 1 U/µg |
| Human Fibroblast Growth Factor-Basic | Invitrogen, Grand Island, NY | 4 ng/mL |
| Insulin-Transferrin- sodium selenite (ITS) media supplement | Roche, Indianapolis, IN | 5 µg/mL Insulin,  5 µg/mL Transferrin,  5 ng/mL Sodium Selenite |
| MgSO4 | Sigma-Aldrich, St. Louis, MO | powder |
| NaCl | Sigma-Aldrich, St. Louis, MO | powder |
| Phosphate-Buffered Saline pH 7.4 (PBS) | Invitrogen, Grand Island, NY | 1X |
| Paraformaldehyde | Sigma-Aldrich, St Louis, MO | 4% |
| PCR Master Mix | ThermoFisher Scientific, K0171, Waltham, MA |  |
| Proteinase Inhibitors | Sigma-Aldrich, St Louis, MO | 10 mM EDTA  10 mM aminocaproic acid  10 mM N-ethylmaleimide  1 mM PMSF |
| RNeasy Mini Kit | Qiagen, Valencia, CA | kit |
| Sulfo-NHS | Sigma-Aldrich, St Louis, MO | 9.2 mg/mL |
| TGFβ1 | Sigma-Aldrich, St Louis, MO | 10 ng/mL |
| TGFβ2 | Sigma-Aldrich, St Louis, MO | 10 ng/mL |
| TGFβ3 | Sigma-Aldrich, St Louis, MO | 10 ng/mL |
| TGFβ1 ELISA Kit | ThermoFisher Scientific, BBS249-4, Waltham, MA | kit |
| TGFβ2 ELISA Kit | R and D Systems, DB250, Minneapolis, MN | kit |
| TGFβ3 ELISA Kit | Abcam, ab272203, Cambridge, MA |  |
| The BCA Protein Assay Kit | Life Technologies, Grand Island, NY | Kit |
| The enzyme-linked immunosorbent HA Quantitative Test Kit | Corgenix, Broomfield CO | Kit |
| Caveolae/Rafts Isolation Kit | Sigma-Aldrich, CS0750, St Louis, MO | Kit |
| NE-PER nuclear and cytoplasmic extraction reagents | ThermoFisher Scientific, BBS249-4, Waltham, MA | Kit |
| Triton X-100 | Sigma-Aldrich, St Louis, MO | 0.2–0.9 mM |
| Western Lighting Chemiluminescence | Perkin Elmer, Waltham, Massachusetts |  |
| Trypsin | Sigma-Aldrich, St Louis, MO | 0.25% |

**Table S2.** Primer and Probe Sequence Use for Quantitative Real-Time PCR.

| **Gene Name** | **Assay ID (TagMan Expression Assay)** |
| --- | --- |
| ATPase | Hs01045013_m1 |
| CA2 | Hs00163869_m1 |
| α-catenin | Hs00972098_m1 |
| β-catenin | Hs00258305_m1 |
| COL4A4 | Hs01011868_m1 |
| GAPDH | Hs02758991_g1 |
| HNK1 | Hs01024500_m1 |
| Keratocan | Hs00559942_m1 |
| KLF4 | Hs00358836_m1 |
| LEF1 | Hs01547250_m1 |
| MSX1 | Hs00427183_m1 |
| N-cadherin | Hs02340558_g1 |
| p120 | Hs00609738_g1 |
| PITX2 | Hs0423069_mH |
| SLC4A4 | Hs01047033_m1 |
| Snail1 | Hs00195591_m1 |
| SOX9 | Hs00165814_m1 |
| TGFβ1 | Hs00998133_m1 |
| TGFβ2 | Hs00234244_m1 |
| TGFβ3 | Hs01086000_m1 |
| TβR1 | Hs00610320_m1 |
| TβR2 | Hs00234253_m1 |
| TβR3 | Hs00234257_m1 |
| ZO1 | Hs00940307_m1 |
|  |  |

**Table S3.** Primary and Secondary Antibodies Used for Immunofluorescence Staining.

| **Primary Antibodies** | | | |
| --- | --- | --- | --- |
| **Antibody** | **Supplier/Catalog (City, State)** | **Source** | **Dilution** |
| α-catenin | Sigma-Aldrich, C2081, St Louis, MO | Rabbit | 1:100 |
| β-catenin | Abcam, ab253431, Cambridge, MA | Rabbit | 1:100 |
| α-CD44 | Thermo-Fisher, Indianapolis, IN | Rat |  |
| CD44 | Abcam, ab254530, Cambridge, MA | mouse |  |
| α-SMA | Sigma-Aldrich, B2261, St Louis, MO | Rabbit | 1:100 |
| ATPase | Cell Signaling, 3010S, Danvers, MA | Rabbit | 1:100 |
| β-actin | Sigma-Aldrich, (Thr 8) St. Louis, MO | Mouse | 1:100 |
| Cav1 | Abcam, ab32577, Cambridge, MA | Rabbit | 1:1000 |
| cJUN1 | Abcam, ab40766, Cambridge, MA | Rabbit | 1:100 |
| pcJUN1 | Cell Signaling, 9261S, Danvers, MA | Rabbit | 1:100 |
| Cyclin D1 | Abcam, ab16663, Cambridge, MA | Rabbit | 1:100 |
| ERK1/2 | Abcam, ab32537, Cambridge, MA | Rabbit | 1:1000 |
| pERK1/2 | Cell Signaling, 4370, Danvers, MA | Rabbit | 1:1000 |
| ERM | Abcam, ab102010, Cambridge, MA | Rabbit | 1:1000 |
| pERM | Cell Signaling, 3141S, Danvers, MA | Rabbit | 1:1000 |
| F-actin | Abcam, ab115777, Cambridge, MA | Rabbit | 1:1000 |
| FAK (Tyr 397) | Cell Signaling, 3283S, Danvers, MA | Rabbit | 1:1000 |
| FAK | Cell Signaling, 3285S, Danvers, MA | Rabbit | 1:1000 |
| γ-secretase (Nicastrin) | Cell Signaling, 5665S, Danvers, MA | Mouse | 1:1000 |
| Keratocan | Thermo-Fisher, PA5-79552, Indianapolis, IN | Rabbit | 1:1000 |
| Hoechst 33342 | Sigma-Aldrich, B2261, St Louis, MO | N/A | 1:500 |
| Histone | Abcam, ab70048, Cambridge, MA | Rabbit | 1:500 |
| Isoantibody | Thermo-Fisher, Isoantibody, Indianapolis, IN |  |  |
| ITIH1 (HC1) | Abcam, ab70048, Cambridge, MA | Mouse | 1:100 |
| MEKK1 | Cell Signaling, 9122S, Danvers, MA | Rabbit | 1:1000 |
| Merlin | Abcam, ab308025, Cambridge, MA | Rabbit | 1:1000 |
| pMerlin | Abcam, ab2478, Cambridge, MA | Mouse | 1:1000 |
| pMEKK1 | Cell Signaling, 4370, Danvers, MA | Rabbit | 1:1000 |
| MLK3 | Cell Signaling, 2817S, Danvers, MA | Rabbit | 1:1000 |
| pMLK3 | Abcam, ab191530, Cambridge, MA | Rabbit | 1:1000 |
| Mouse IgG | Agilent Dako, X0910, Santa Clara, CA | - | 1:100 |
| MT1MMP | Abcam, ab76011, Cambridge, MA | Rabbit | 1:1000 |
| MT1MMP, Catalytic Domain | Sigma-Aldrich, MAB1767,St. Louis, MO | MAB1767 | 1:1000 |
| N-cadherin | Abcam, ab76011, Cambridge, MA | Rabbit | 1:1000 |
| p38 | Abcam, ab170099, Cambridge, MA | Rabbit | 1:1000 |
| pp38 | Cell Signaling, 4511S, Danvers, MA | Rabbit | 1:1000 |
| p75 | Abcam, ab52987, Cambridge, MA | Rabbit | 1:1000 |
| p120 | Abcam, ab17969, Cambridge, MA | Rabbit | 1:1000 |
| PI3K | Cell Signaling, 4292, Danvers, MA | Rabbit | 1:1000 |
| pPI3K | Cell Signaling/4288, Danvers, MA | Rabbit | 1:1000 |
| PTX3 | Enzo Life Science, ALX-210-365, Farmingdale, NY | Rabbit | 1:100 |
| pSMAD2/3 | Sigma-Aldrich,St. (Thr 8) Louis, MO | Rabbit | 1:100 |
| pSMAD1/5 | Santa Cruz (Ser 463/Ser 465), Santa Cruz, CA | Goat | 1:50 |
| Rabbit IgG | Agilent Dako, X0902, Santa Clara, CA | - | 1:100 |
| S100A4 | Abcam, ab290059, Cambridge, MA | HEK 293 cells | 1:100 |
| TAK1 | Abcam, ab109526, Cambridge, MA | Rabbit | 1:1000 |
| pTAK1 | Cell Signaling, 4531, Danvers, MA | Rabbit | 1:1000 |
| Transferrin | Abcam, ab214039, Cambridge, MA | Rabbit | 1:1000 |
| TGFβ1 | Abcam, ab215715, Cambridge, MA | Rabbit | 1:1000 |
| TGFβ2 | Abcam, ab238249, Cambridge, MA | Goat | 1:1000 |
| TGFβ3 | Abcam, ab227711, Cambridge, MA | Rabbit | 1:1000 |
| TβR1 | Abcam, ab235578, Cambridge, MA | Rabbit | 1:1000 |
| TβR2 | Cell Signaling, 79429, Danvers, MA | Rabbit | 1:1000 |
| TβR3 | Abcam, ab166705, Cambridge, MA | Mouse | 1:1000 |
| ZO1 | Abcam, ab216880, Cambridge, MA | Rabbit | 1:1000 |
|  |  |  |  |
| **Secondary Antibodies** | | | |
| **Antibody** | **Supplier** | **Source** | **Dilution** |
| Alexa Fluor 633 Anti-Goat | Thermo Fisher Scientific, Indianapolis, IN | Donkey | 1: 100 |
| Alexa Fluor 488 Anti-Mouse | Thermo Fisher Scientific, Indianapolis, IN | Donkey | 1: 100 |
| Alexa Fluor 555 Anti-Rabbit | Thermo Fisher Scientific, Indianapolis, IN | Donkey | 1: 100 |
| Alexa Fluor 633 Anti-Chicken | Thermo Fisher Scientific, Indianapolis, IN | Goat | 1: 100 |
| Anti-mouse | Abcam, ab97057, Cambridge, MA | Goat | 1:1000 |
| Anti-rabbit | Abcam, ab6728, Cambridge, MA | Rabbit | 1:1000 |
| Anti-rat | Abcam, ab6721, Cambridge, MA | Goat | 1:1000 |

**Quality Control**

HC-HA/PTX3 Preparations

Each batch of HC-HA/PTX3 was purified from AM extract obtained from 6 pooled donors undergoing four successive runs of CsCl-density gradient ultracentrifugation by pooling fractions that contains HA but not proteins, determined by the HA quantitative ELISA and BCA, respectively. In addition, each batch of HC-HA/PTX3 was released after passing the potency assay with the acceptance criteria of no less than 89% inhibition of tartrate-resistant acid phosphatase (TRAP) activity of osteoclast differentiation in cloned monocytes of murine RAW264.7 cell line (ATCC; Manassas, VA) by receptor activator of nuclear factor kappa-Β ligand (PeproTech; Cranbury, NJ). Furthermore, Western blot analysis using respective antibody specific to HC1 and PTX3 with or without hyaluronidase (HAase) digestion and with or without reduction by DTT confirmed the presence of HC1-HA complex containing HMW PTX3 that was reduced to dimer and monomer.

Quality Control Procedures Implemented During Amniotic Membrane Processing

Additional quality control measures have been taken by Statistical Control Chart of 23 consecutive batches of HC-HA/PTX3 manufactured from June 2018 to June 2021. Our results showed that if we set the pre-specified acceptance criteria for not exceeding 50 μg/mL, i.e., the upper control limit (UCL), for the HA concentration, all HC-HA/PTX3 lots yielded a percent inhibition from 86% to 100% in the ODI-TRAP potency assay. Western blot analysis of all HC-HA/PTX3 lots showed an 80 kDa HC1 band and a high molecular weight (HMW) PTX3 smear after hyaluronidase digestion. After additional reduction with 40 mM dithiothreitol (DTT) at an alkaline pH of 8.8, HMW PTX3 smear yielded a PTX3 dimer (~ 90 kDa) and monomer (~ 45 kDa). These results remained consistent among the 23 lots of HC-HA/PTX3 analyzed.
